# AWARENESS OF PERSONAL GAINS, MINDFULNESS, AND PSYCHOLOGICAL WELLBEING AMONG DEMENTIA CAREGIVERS

**DOI:** 10.1093/geroni/igae098.4125

**Published:** 2024-12-31

**Authors:** Darby Simon, Evan Plys, Elizabeth Allen, Morgan Seward, Ana-Maria Vranceanu

**Affiliations:** Massachusetts General Hospital, Boston, Massachusetts, United States; Massachusetts General Hospital, Boston, Massachusetts, United States; Massachusetts General Hospital, Boston, Massachusetts, United States; Massachusetts General Hospital, Boston, Massachusetts, United States; Massachusetts General Hospital, Boston, Massachusetts, United States

## Abstract

The current study tested if increased awareness of personal gains from caregiving and mindfulness skills each independently related to psychological well-being among caregivers of people living with dementia, beyond caregiver role-related factors. We conducted a secondary analysis of baseline data from 92 stressed caregivers, aged 22 to 83 years old (M=53.90, SD= 14.53), who participated in a clinical trial. Three separate multiple regression models tested the relationship between awareness of personal gains (Caregiver Reaction Scale- Personal Gain subscale), mindfulness (Applied Mindfulness Process Scale), and outcomes of depression (Hospital Anxiety and Depression Scale [HADS]-Depression), stress (Perceived Stress Scale-10), and anxiety (HADS-Anxiety), while controlling for role overload, caregiving competence, and hours of providing direct care per week. Results revealed that, in the full model, higher awareness of personal gains (β = -.31, p <.001) was significantly related to lower levels of depression. Higher awareness of personal gains (β = -.23, p <.05) and higher levels of mindfulness (β = -.18, p <.05) were significantly related to lower levels of stress. Neither awareness of personal gains nor mindfulness predicted caregivers’ anxiety. These findings provide evidence that awareness of personal gains and mindfulness uniquely predict aspects of dementia caregivers’ psychological well-being, even after controlling for well-established predictors. Psychological interventions may target caregivers’ awareness of their personal gains and mindfulness to improve their caregiving experience and psychological health. Anxiety may require different treatment approaches.

